# Sexually Explicit Internet Material and Adolescents’ Sexual Uncertainty: The Role of Disposition-Content Congruency

**DOI:** 10.1007/s10508-015-0594-1

**Published:** 2015-09-15

**Authors:** Johanna M. F. van Oosten

**Affiliations:** The Amsterdam School of Communication Research, ASCoR, University of Amsterdam, PO Box 15791, 1001 NG Amsterdam, The Netherlands

**Keywords:** Internet, Media effects, Adolescents

## Abstract

Previous research has suggested that adolescents’ exposure to sexually explicit internet material (SEIM) may result in sexual uncertainty because the content of SEIM may conflict with what adolescents have learned about sex. However, research on which type of adolescent is most susceptible to the relation between SEIM use and sexual uncertainty is lacking. This study therefore investigated whether the relationship between SEIM use and sexual uncertainty depends on within-gender differences in sexual dispositions (i.e., impersonal sex orientation and hypergendered orientation). Using data from a representative two-wave panel survey among 1765 Dutch adolescents (aged 13–17), I found that SEIM use predicted sexual uncertainty only among girls with a low hypergendered orientation and girls with a relatively high impersonal sex orientation.

## Introduction

Sexually explicit internet material (SEIM) is increasingly considered an influence on adolescent sexuality, given its high amount of sexual content and the high number of adolescents who encounter such material online (for a review, see Owens, Behun, Manning, & Reid, 2012). About 93 % of boys and 62 % of girls have encountered SEIM before the age of 18 (Sabina, Wolak, & Finkelhor, 2008). Moreover, in a representative US survey, 34 % of adolescents between the ages of 13 and 17 reported that they had deliberately watched SEIM, often out of sexual curiosity (Wolak, Mitchell, & Finkelhor, 2007). SEIM is typically defined as material on or from the internet that is intended to arouse the recipient, including the explicit, unconcealed depiction of (aroused) genitals and sexual activities, such as oral sex and anal or vaginal penetration (Peter & Valkenburg, 2009).

Several scholars have pointed out that the sexual content that adolescents encounter may conflict with beliefs about sexuality that adolescents have adopted from families, schools, and peers (e.g., Arnett, 1995; Thornburgh & Lin, 2002; Wolak et al., 2007). In this context, researchers have recently started to pay attention to the *sexual uncertainty hypothesis* (Sparks, 2013). According to this hypothesis, adolescents will react with sexual uncertainty when they are confronted with sexual material, such as SEIM, that is in conflict with their sexual socialization (Peter & Valkenburg, 2008, 2010). Sexual uncertainty refers to being unclear about one’s sexual beliefs and values, and may show in poorly integrated, unclearly defined, and temporally unstable sexual beliefs (Peter & Valkenburg, 2008).

Although the sexual uncertainty hypothesis has initially been supported empirically (Peter & Valkenburg, 2008, 2010), the relationship between SEIM use and sexual uncertainty is still understudied. In particular, it is unclear which types of adolescents are most susceptible to the influence of SEIM on sexual uncertainty. Previous research on the sexual uncertainty hypothesis has focused on differences between boys and girls (i.e., between-gender differences), assuming that girls are expected to experience the largest clash between the content in SEIM and their gender-specific sexual socialization. Results on such between-gender differences, however, have been inconsistent (Peter & Valkenburg, 2010). One potential explanation for these inconsistencies is that it may not be sufficient to look at between-gender differences as not all girls or boys are the same.

Recent media effects models have emphasized congruency effects between content and individual dispositions that vary *within* gender, such as attitudes and beliefs (Valkenburg & Peter, 2013). This focus in recent theorizing merges with research on the effects of sexually explicit material among adults, which have consistently been found to depend on (within-gender) differences in sexual dispositions (e.g., Kingston, Malamuth, Fedoroff, & Marshall, 2009; Malamuth, Addison, & Koss, 2000). However, such within-gender differences in effects of sexual content have not been investigated among adolescents. Since forming a stable sense of a sexual self is one of the main tasks of adolescence (Steinberg, 2008), it is not only important to know that SEIM use can hinder this task by increasing sexual uncertainty, but—even more importantly—to also know which type of adolescent is most susceptible to this influence of SEIM. The present study therefore aimed to investigate for which type of adolescents the relation between SEIM use and adolescents’ sexual uncertainty occurs, focusing on within-gender differences in impersonal sex orientation and hypergendered orientation.

### Gender Differences in Sexual Socialization

Typically, researchers have proposed that whether adolescents experience congruency with SEIM depends on adolescents’ gender (Peter & Valkenburg, 2010): As SEIM seems more congruent with male sexual socialization than with female sexual socialization (Peter & Valkenburg, 2010), girls may react with more sexual uncertainty to SEIM than boys do. This expectation is based on the social construction-of-sexuality perspective, which states that male and female adolescents undergo a different sexual socialization (see for instance, Bohan, 1993; Foucault, 1978; Gagnon & Simon, 1973). For instance, sexual behavior is generally guided by the sexual double standard, in that having sex outside of a committed relationship is still more acceptable for boys than for girls (e.g., Allen et al., 2007; Fugère, Escoto, Cousins, Riggs, & Haerich, 2008; Petersen & Hyde, 2010, 2011). Similarly, girls are usually not expected to act sexually or act on their sexual impulses, whereas boys are typically allowed, or sometimes even expected, to initiate sex and to be sexually dominant (e.g., Allen et al., 2007; Tolman, 2002). Rather, girls are taught to be sexy in order to attract men, but are frequently discouraged to be sexual and have sexual desires of their own (Tolman, 2002; Zubriggen et al., 2007). Finally, sexual desire outside a committed relationship—including sexual desire as a reaction to sexually explicit material—tends to be less accepted for females than for males (Allen et al., 2007; Petersen & Hyde, 2010, 2011).

As sexually explicit material relatively often depicts casual sex, male sexual dominance, and women who are willing and desiring to have sex (for content analyses, see: Bridges, Wosnitzer, Scharrer, Sun, & Liberman, 2010; Brosius, Weaver, & Staab, 1993; Dines, Jensen, & Russo, 1998), the content in SEIM may be somewhat more congruent with male socialization than with female socialization. In fact, previous research on responses to sexually explicit material has shown that women are generally more critical toward such content than men because such content is not congruent with women’s sexual socialization (Allen et al., 2007; Laan, Everaerd, van Bellen, & Hanewald, 1994; Mosher & Maclan, 1994).

At the same time, gender differences in sexual attitudes and behavior are generally small and have been decreasing in the past decades (Petersen & Hyde, 2010, 2011). For instance, even though men are still slightly more likely to accept casual sex than women, women have adopted more permissive attitudes toward sex over the past decades (Fugère et al., 2008; Petersen & Hyde, 2010, 2011). It has also been argued that there are more similarities than differences in psychological variables, including sexuality-related dispositions, between males and females (i.e., the gender similarities hypothesis, Hyde, 2005). Moreover, there seem to be differences among girls and women in the extent to which they adhere to the societal standards about female sexuality (e.g., Milnes, 2004; Renold & Ringrose, 2011; Vanwesenbeeck, 2009). For instance, some women seem to consider self-sexualizing and sexually loose behaviors more enjoyable and rewarding than others (Liss, Erchull & Ramsey, 2011).

These findings may imply that not all girls respond in the same way to sexually explicit material and that, in some instances, girls may not differ from boys in their responses to such material. In fact, research has shown that women with similar expectations about sex as men did not differ from men in their responses to sexually explicit material (Mosher & Maclan, 1994). Similarly, women have been shown to differ in their reactions (i.e., discomfort and ego-hurting versus appreciation and ego-boosting) to explicit sex on television, depending on their sexual self-image (Vanwesenbeeck, 2001). Moreover, gender differences in sexual attitudes and behavior have been found to disappear when taking into account individual difference variables such as age, educational level, and religiosity, but also sex-related motives, attitudes, and beliefs (Vanwesenbeeck, 2009). In line with these findings, previous research on the relationship between the use of SEIM and adolescents’ sexual uncertainty did not find gender differences in this relationship (Peter & Valkenburg, 2010). It thus stands to reason that other variables than gender may be at play when explaining adolescents’ susceptibility to the effects of SEIM.

### Individual Dispositions and the Congruency with SEIM

According to the Differential Susceptibility to Media Effects Model (DSMM, Valkenburg & Peter, 2013), effects of media use can also depend on pre-existing differential susceptibility variables, including dispositional susceptibility variables such as personality, cognitions, values, attitudes, and beliefs (Valkenburg & Peter, 2013). These variables may differ just as much within gender as they do between genders. The lack of between-gender differences in previous research on the relationship between SEIM use and sexual uncertainty may thus be the result of the variance in dispositional susceptibility among adolescents.

The way in which dispositional susceptibility variables affect the relationship between the use of SEIM and sexual uncertainty is specified within the DSMM by the *disposition*-*content congruencyhypothesis*. The disposition-content congruency hypothesis generally posits that media effects depend on the congruency between media content and one’s dispositions. Specifically, the model predicts that media content that matches one’s dispositions (i.e., congruent media content) reinforces existing mental schemata (Valkenburg & Peter, 2013). Although not explicitly predicted in the DSMM, disposition-content congruency effects may also imply that when media content does not match one’s dispositions, existing schemata may be challenged. Consequently, it can be expected that being exposed to content in SEIM that is incongruent with adolescents’ dispositions may reduce the certainty with which adolescents hold their sexual beliefs and values.

In line with the notion of disposition-content congruency effects, research on the confluence model (Kingston et al., 2009; Malamuth et al., 2000; Malamuth, Hald, & Koss, 2012) has suggested that effects of sexually explicit material on adult men specifically depend on the congruency between such material and sexual dispositions that differ among men. In terms of these dispositions, the confluence model has particularly focused on men’s impersonal and hypergendered orientations toward sex (e.g., Malamuth et al., 2000, 2012). An impersonal sex orientation refers to the degree to which one believes that sexual relations without emotional bonding and relational commitment are acceptable and pleasurable (Malamuth et al., 2000; Malamuth, Linz, Heavey, Barnes, & Acker, 1995). A hypergendered orientation encompasses the *hypermasculinity* concept for men and the *hyperfemininity* concept for women (Hamburger, Hogben, McGowan, & Dawson, 1996; Kreiger & Dumka, 2006). Hypermasculinity refers to men’s tendency to engage in hostile and dominant behavior (Mosher & Sirkin, 1984). Hyperfemininity refers to women’s acceptance of female objectification and male dominance, and the importance of being physically attractive in order to attract men (Murnen & Byrne, 1991).

The relevance of impersonal sex orientation and hypergendered orientation for disposition-content congruency effects is also supported by content analyses that point to the congruency between the content in SEIM and these sexual dispositions. In line with impersonal sex orientation, sexually explicit material depicts sex as occurring predominantly between uncommitted partners, with women typically being portrayed as easily available (Brosius et al., 1993; Ertel, 1990; Klaassen & Peter, 2014). Corresponding with hypergendered orientation, male sexual dominance and female sexual subordination, and the importance for women to be sexually attractive for men are frequently featured in sexually explicit material (Bridges et al., 2010; Brosius et al., 1993; Cowan & Campbell, 1994; Gorman, Monk-Turner, & Fish, 2010; Klaassen & Peter, 2014).

In an extension of the confluence model to non-aggressive explicit sexual media content among women, a recent study has found that women with an impersonal sex orientation evaluated a person engaging in casual sex more positively than did women without an impersonal sex orientation (Boot, Peter, & van Oosten, 2014). First evidence has also emerged that women with a hypergendered orientation respond less critically to sexually explicit material than women who do not have a hypergendered orientation (van Oosten, Peter, & Boot, 2015). These findings thus suggest that adolescents’ impersonal sex orientation and hypergendered orientation are important susceptibility variables in the relationship between SEIM use and sexual uncertainty and may explain disposition-content congruency effects beyond gender differences in sexual socialization.

In conclusion, given frequent themes in SEIM and previous research results, adolescents’ high levels of impersonal sex orientation and hypergendered orientation can be expected to be congruent with the content of SEIM. Conversely, low levels of impersonal sex orientation and hypergendered orientation are likely to be incongruent with the content in SEIM. Extending previous predictions that the relationship between SEIM use and sexual uncertainty would only hold for girls (Peter & Valkenburg, 2010), I therefore expected that the lack of congruency between sexual content and impersonal sex orientation and hypergendered orientation would further boost the relationship between SEIM use and sexual uncertainty for girls. Specifically, as girls with low levels of impersonal sex orientation and hypergendered orientation are most likely to experience incongruence between SEIM and their dispositions, they are most likely to respond with sexual uncertainty to SEIM use. In contrast, as girls with high impersonal sex orientation and hypergendered orientation likely experience some congruency between SEIM and their sexual dispositions, they are not expected to respond with sexual uncertainty. More specifically, I hypothesized:

#### **H1**

SEIM use will be associated with sexual uncertainty among girls with (a) a low impersonal sex orientation and (b) a low hypergendered orientation, as opposed to girls with a high impersonal sex orientation and a high hypergendered orientation.

It is important to note that this hypothesis also implies that the previously predicted between-gender differences depend on within-gender differences in sexual dispositions, such that girls are only expected to differ from boys when they have low levels of impersonal sex orientation and hypergendered orientation.

## Method

### Sample and Procedure

I analyzed data from a three-wave longitudinal panel survey that was conducted among a nationally representative sample of Dutch adolescents (aged 13–17; 50 % male) in May and June 2013 (Wave 1), November and December 2013 (Wave 2), and May and June 2014 (Wave 3) by Veldkamp, a Dutch survey institute. Because only two time points were needed to investigate the longitudinal associations between SEIM use and sexual uncertainty adequately, I focused on the first two waves of the survey. Moreover, I focused on the first two waves because this would allow me to conduct the analyses on a larger and more representative sample (due to panel attrition in the second and third waves). Respondents were randomly selected from a pool of respondents, which was originally sampled randomly among the Dutch population and is continuously updated. Unlike in many online access panels, the sample thus does not suffer from snowballing effects in the sampling process and self-selection biases in the survey. The response rate of the first wave was 78 %, and the response rate of the second wave was 83 %, resulting in a final sample of 1765 participants. Of the sample, 93.3 % had a heterosexual orientation (i.e., were solely attracted to members of the opposite sex). As for the sexual experience of the sample, 26 % had engaged in genital touching, 14 % had engaged in oral sex, and 15 % had engaged in sexual intercourse at Wave 1. More than half (55.5 %) of the sample belonged to the highest and second highest level of socio-economic status (SES, based on the occupation and educational level of the parents of the participants). The lowest and second lowest SES level included 45.5 % of the sample. This is similar to other research in the Netherlands showing that the division of higher and lower SES is approximately 50/50 (Hulshof, Brussaard, Kruizinga, Telman, & Löwik, 2003).

Ethical approval from the University of Amsterdam, as well as informed consent of the adolescents’ parents, was obtained before the start of the study. Respondents were asked to complete an online survey at home. For sensitive issues such as sexuality, online surveys have been shown to be a useful alternative to other survey modes (Mustanski, 2001). Respondents were notified that the study was about sexual issues, that they could stop at any time they wished, and that the principal investigators could not trace identifying information. After completion of each wave, the respondents received a voucher worth five Euro.

### Measures

With the exception of SEIM use, the variables in this study were measured on 7-point scales ranging from 1 (*agree entirely*) to 7 (*disagree entirely*). Items were recoded such that higher scores indicated higher scores of each variable. In the questionnaire, the order of items was randomized.

#### SEIM Use

I used a measure of SEIM use that had shown to be a valid and reliable measure in earlier studies on the relationship between SEIM use and sexual uncertainty (Peter & Valkenburg, 2008, 2010). Respondents were asked to indicate how often in the previous 6 months they had intentionally looked at sexual content on their computer, either online or offline (i.e., downloaded material), and in a separate question how often they encountered such content accidentally. Respondents were notified that the question was about pornographic internet material, not nudity. Sexual content was specified as (a) pictures with clearly exposed genitals, (b) movies with clearly exposed genitals, (c) pictures in which people were having sex, and (d) movies in which people were having sex. For each type of sexual content, the response categories ranged from 1 (*several times a day*) to 7 (*never*). Items were recoded so that higher scores indicated more frequent use of SEIM. The items for intentional use formed a unidimensional scale with an explained variance higher than 88 % and a Cronbach’s α .96 in both waves (*M* = 1.77, *SD* = 1.35 in Wave 1; *M* = 1.77, *SD* = 1.28 in Wave 2). The items for accidental use also formed a unidimensional scale with an explained variance higher than 85 % and a Cronbach’s α higher than .94 in both waves (*M* = 1.85, *SD* = 1.14 in Wave 1; *M* = 1.90, *SD* = 1.17 in Wave 2). Intentional and accidental SEIM use were highly correlated, *r* = .75, *p* < .001, in Wave 1 and *r* = .71, *p* < .001 in Wave 2.

#### Sexual Uncertainty

I used a six-item measure of sexual uncertainty that was developed to measure the extent to which adolescents are unclear about their sexual beliefs and values (Peter & Valkenburg, 2008, 2010). An example item is “As far as sex is concerned I am not sure about what I like and what I dislike.” In both waves, the items loaded on one factor (explained variance >70 %) and formed a reliable scale (Cronbach’s alpha >.85; *M* = 3.13, *SD* = 1.34 in Wave 1; *M* = 3.04, *SD* = 1.33 in Wave 2).

#### Hypergendered Orientation

The hypergendered orientation measure was based on items from the Hyperfemininity Scale (Murnen & Byrne, 1991) for girls and on the Hypermasculinity Index (Mosher & Sirkin, 1984) for boys. I took the 6 items with the highest corrected item-total correlations from a previous pilot study among female (*N* = 77) and male (*N* = 36) undergraduate students, and changed the forced-choice format of the items into a Likert-scale format to increase variance and the ability to discriminate within levels of hypergendered orientation (cf. Hamburger et al., 1996). When necessary, original items with an adult bias were modified into items appropriate for adolescents, retaining their original meaning.

Example items for female respondents are “It’s ok if a boy acts a little dominant towards me” and “When you act sexy, you will get guys to do what you want.” In both waves, the hyperfeminine items loaded on one factor (explained variance >55 %), and formed a reliable scale (Cronbach’s alpha >.83, *M* = 3.53, *SD* = 1.26 in Wave 1; *M* = 3.46, *SD* = 1.32 in Wave 2). Example items for male respondents are “Those who can, fight. Those who can’t fight, run away” and “People sometimes tell me I take stupid risks.” The hypermasculine items loaded on one factor (explained variance >54 %), and formed a reliable scale in both waves (Cronbach’s alpha >.83, *M* = 3.39, *SD* = 1.32 in Wave 1; *M* = 3.33, *SD* = 1.29 in Wave 2). The measures of hyperfeminine orientation and hypermasculine orientation (Wave 1) were both significantly (*all p’s* ≤ .001)—and in similar ways—related to the other main variables in the study and were therefore combined in one hypergendered orientation score (*M* = 3.46, *SD* = 1.29 in Wave 1; *M* = 3.39, *SD* = 1.31 in Wave 2).

#### Impersonal Sex Orientation

I used an adjusted version of the Sociosexual Orientation Inventory (SOI, Simpson & Gangestad, 1991) that had been used as a measure of impersonal sex orientation in previous research (Boot et al., 2014; Jacques-Tiura, Abbey, Parkhill, & Zawacki, 2007). The three items from the attitudinal subscale of the SOI (Penke & Asendorpf, 2008) were changed into four items, which were adjusted somewhat to make them more suitable for adolescents. Example items are “It is okay to hook-up with different people at a time” and “One-night stands can be very enjoyable.” The items loaded on one factor (explained variance >74 %) showed good internal consistency (*α* > .88), and were therefore averaged to form the impersonal sex orientation scale (*M* = 2.24, *SD* = 1.28 in Wave 1; *M* = 2.29, *SD* = 1.34 in Wave 2).

### Control Variables

I controlled for the following variables that had been shown to influence sexual uncertainty in previous research as well as in the current data: age (Hensel, Fortenberry, O’Sullivan, & Orr, 2011), religiosity (McMillen, Helm, & McBride, 2011), sexual experience (Lindgren, Schacht, Mullins, & Blayney, 2011), and social comparison orientation (VanYperen & Buunk, 1991). Age was measured in years (*M* = 14.95, *SD* = 1.41, in Wave 1). Religiosity was measured with the items “I am religious” and “My faith is important to me”, on a scale from 1 (*does not apply at all*) to 7 (*fully applies to me*) (*r* = .92, *M* = 2.71, *SD* = 1.93). To measure sexual experience, respondents were asked to answer whether they had experience with the following sexual behaviors: (a) touching each others’ genitals, (b) giving or receiving oral sex, and (c) intercourse, which was coded as 1 (*yes*) or 0 (*no*) (α = .87, *M* = .14, *SD* = .26). Social comparison orientation was measured with four items with the highest factor loadings (on scale Factor 1) from the Iowa-Netherlands Comparison Orientation Measure (Gibbons & Buunk, 1999), e.g., “I always pay a lot of attention to how I do things compared to how others do things”, on a scale from 1 (*does not apply at all*) to 7 (*fully applies to me*) (α = .87, *M* = 4.33, *SD* = 1.29).

### Data Analysis

I tested the hypotheses by analyzing three-way interactions between SEIM use, gender, and hypergendered orientation as well as between SEIM use, gender, and impersonal sex orientation, in an OLS regression analysis with sexual uncertainty (Wave 2) as the dependent variable. The hypotheses were tested with intentional SEIM use. Additional analyses were conducted for accidental SEIM use for comparison purposes. I controlled for the following variables (all measured at Wave 1): age, religiosity, sexual experience, social comparison orientation, SEIM use, gender, hypergendered orientation, impersonal sex orientation, and sexual uncertainty. In addition, I controlled for the following lower order (i.e., two-way) interactions; SEIM use × gender, SEIM use × impersonal sex orientation, SEIM use × hypergendered orientation, impersonal sex orientation × gender, and hypergendered orientation × gender. Post hoc probing of the three-way interaction was done using simple slope analyses as well as slope difference tests (Dawson & Richter, 2006). All variables were mean centered before the regression analyses to reduce scale invariance and multicollinearity (Aiken & West, 1991).

## Results

**Fig. 1:**
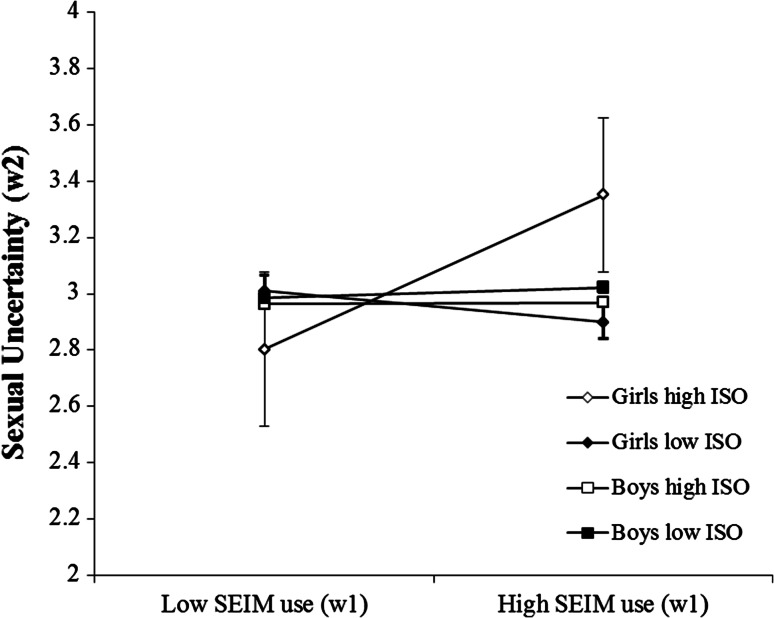
The relationship between intentional SEIM use and sexual uncertainty, for low (−1 SD from the mean) and high (+1 SD from the mean) impersonal sex orientation (ISO) scores for boys and girls. Low and high SEIM use refer to frequency scores of intentional SEIM use of 1 SD below and 1 SD above the mean

Zero-order correlations for the full sample are shown in Table 1, and for boys and girls separately in Table 2. Regression coefficients for all the variables in the analysis are shown in Table 3. The results showed a significant three-way interaction between intentional SEIM use (Wave 1), gender and impersonal orientation (Wave 1) on sexual uncertainty (Wave 2), *β* = 0.10, *B* = 0.11, *SE* = 0.06, *p* = .04. The regression coefficients indicated a positive influence of SEIM on sexual uncertainty for girls, and this influence became even stronger as impersonal sex orientation increased (see Table 3). To test H1a rigorously, post-hoc analyses on the three-way interactions were conducted (see Fig. 1; Table 4). Specifically, I conducted simple slope analyses for girls (and boys) with high (1 SD above the mean) and low (1 SD below the mean) levels of impersonal sex orientation (see Table 4 for simple slope coefficients). In contrast to Hypothesis 1a, I only found a significant relationship between SEIM use and sexual uncertainty for girls with *high* levels of impersonal sex orientation. That is, for girls with levels of impersonal sex orientation one standard deviation above the mean (i.e., a score of 3.52), sexual uncertainty increased by .23 when SEIM use increased by one unit. The slope difference test showed that the within-gender difference between the regression slopes for girls with a low and a high impersonal orientation was significant, *t*(1,748) = 2.07, *p* = .039, Cohen’s *d* = .10.

**Table 1:** Zero-order correlations between the variables, for the full sample

	1	2	3	4	5	6	7	8	9
1. Intentional SEIM use (w1)									
2. Impersonal sex orientation (w1)	0.44***								
3. Hypergendered orientation (w1)	0.22***	0.40***							
4. Sexual uncertainty (w2)	0.04	0.11***	0.14***						
*Control variables* (*at Wave 1*)									
5. Age	0.12***	0.10***	0.11***	−0.03					
6. Sexual experience	0.20***	0.22***	0.25***	−0.07**	0.42***				
7. Religiosity	−0.06*	−0.13***	−0.03	−0.08**	−0.01	−0.10***			
8. Social comparison orientation	0.09***	0.12***	0.26***	0.14***	0.06**	0.02	0.07**		
9. Gender	−0.34***	−0.27***	0.05*	−0.03	0.08**	0.03	0.07**	0.10***	
10. Sexual uncertainty	0.10***	0.24***	0.20***	0.38***	−0.04	−0.07**	−0.07**	0.18***	−0.09***

*SEIM* Sexually explicit internet material, *w1* Wave 1, *w2* Wave 2

* *p* < .05; ** *p* < .01; *** *p* < .001

**Table 2:** Zero-order correlations between the variables, for boys and girls separately

	1	2	3	4	5	6	7	8
*Boys*								
1. Intentional SEIM use (w1)								
2. Impersonal sex orientation (w1)	0.43***							
3. Hypergendered orientation (w1)	0.28***	0.42***						
4. Sexual uncertainty (w2)	0.04	0.07*	0.11**					
*Control variables* (*at Wave 1*)								
5. Age	0.21***	0.15***	0.04	−0.01				
6. Sexual experience	0.26***	0.28***	0.26***	−0.08*	0.38***			
7. Religiosity	−0.02	−0.07*	−0.01	−0.07*	0.01	−0.04		
8. Social comparison orientation	0.15***	0.13***	0.18***	0.12***	0.03	0.02	0.08*	
9. Sexual uncertainty	0.08*	0.18***	0.12**	0.36***	−0.04	−0.10**	0.03	0.16***
*Girls*								
1. Intentional SEIM use (w1)								
2. Impersonal sex orientation (w1)	0.29***							
3. Hypergendered orientation (w1)	0.22***	0.45***						
4. Sexual uncertainty (w2)	0.04	0.15***	0.17***					
*Control variables* (*at Wave 1*)								
5. Age	0.06	0.11**	0.16***	−0.04				
6. Sexual experience	0.20***	0.20***	0.24***	−0.05	0.45***			
7. Religiosity	−0.07*	−0.16***	−0.05	−0.08*	−0.03	−0.15***		
8. Social comparison orientation	0.12***	0.19***	0.34***	0.16***	0.08*	0.01	0.05	
9. Sexual uncertainty	0.08*	0.30***	0.30***	0.40***	−0.02	−0.04	−0.10**	0.23***

*SEIM* Sexually explicit internet material, *w1* Wave 1, *w2* Wave 2

* *p* < .05; ** *p* < .01; *** *p* < .001

**Table 3:** Regression coefficients for the prediction of sexual uncertainty (Wave 2)

	*B*	*SE*	*p* value
Intentional SEIM use (w1)	0.05	0.05	0.274
Gender	0.03	0.08	0.688
ISO (w1)	0.02	0.03	0.592
HGO (w1)	0.04	0.03	0.226
Sexual uncertainty (w1)	0.34	0.02	0.001
Age (w1)	−0.01	0.02	0.846
Sexual experience (w1)	−0.32	0.13	0.017
Religiosity (w1)	−0.05	0.02	0.004
Social comparison orientation (w1)	0.07	0.02	0.006
Intentional SEIM use × gender	0.08	0.09	0.369
Intentional SEIM use × ISO	0.05	0.03	0.070
Intentional SEIM use × HGO	−0.10	0.04	0.007
Gender × ISO	0.06	0.06	0.302
Gender × HGO	−0.10	0.06	0.105
Intentional SEIM use × gender × ISO	0.11	0.06	0.045
Intentional SEIM use × gender × HGO	−0.15	0.07	0.045

*SEIM* Sexually explicit internet material, *ISO* impersonal sex orientation, *HGO* hypergendered orientation, *w1* Wave 1

**Table 4:** Simple slope coefficients of the relationship between intentional SEIM use (Wave 1) and sexual uncertainty (Wave 2) for boys and girls with low and high levels of impersonal sex orientation (ISO) and hypergendered orientation (HGO)

	*B*	*t*(1,748)
Girls high ISO	0.23*	2.11
Girls low ISO	−0.05	−0.44
Boys high ISO	0.00	0.07
Boys low ISO	0.02	0.29
Girls high HGO	−0.13	−1.63
Girls low HGO	0.32*	2.04
Boys high HGO	−0.02	−0.48
Boys low HGO	0.04	0.92

* *p* < .05

**Fig. 2:**
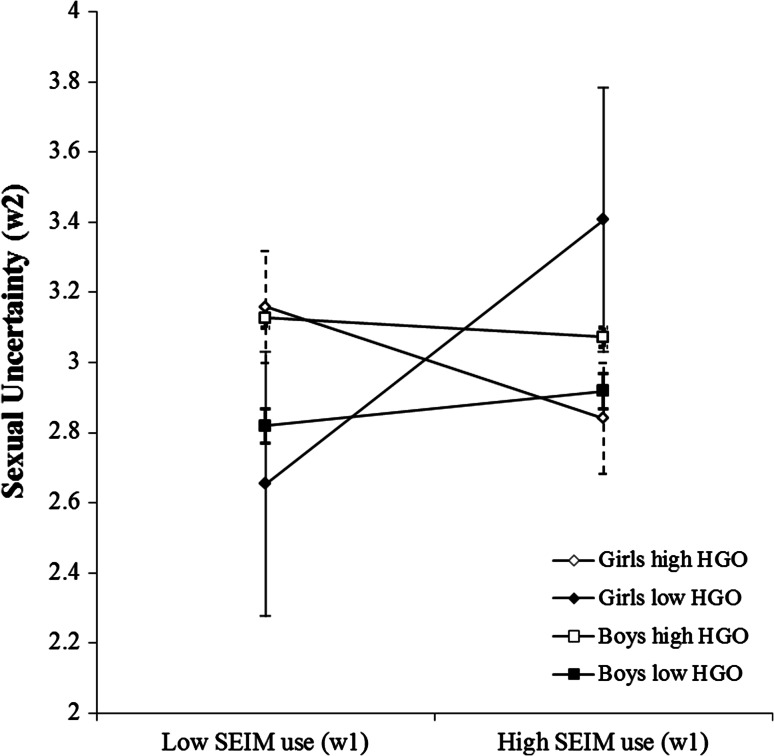
The relationship between intentional SEIM use and sexual uncertainty, for low (−1 SD from the mean) and high (+1 SD from the mean) hypergendered orientation (HGO) scores for boys and girls. Low and high SEIM use refer to frequency scores of intentional SEIM use of 1 SD below and 1 SD above the mean

In line with Hypothesis 1b, I found a significant three-way interaction between intentional SEIM use (Wave 1), gender and hypergendered orientation (Wave 1) on sexual uncertainty (Wave 2), *β* = −0.10, *B* = −0.15, *SE* = 0.07, *p* = .04. The regression coefficients showed a positive influence of SEIM on sexual uncertainty for girls, and this influence became stronger as hypergendered orientation decreased (see Table 3). The three-way interaction suggested that the positive relationship between SEIM use and sexual uncertainty would hold only for girls with a low hypergendered orientation, as predicted in H1b (see Fig. 2; Table 4). I conducted simple slope analyses for girls (and boys) with high (1 SD above the mean) and low (1 SD below the mean) levels of hypergendered orientation (see Table 4 for simple slope coefficients). That is, for girls with levels of hypergendered orientation one standard deviation below the mean (i.e., a score of 2.17), sexual uncertainty increased by .32 when SEIM use increased by one unit. The slope difference test showed that the within-gender difference between the regression slopes for girls with a low and a high hypergendered orientation differed was significant, *t*(1,748) = −2.47, *p* = .01, Cohen’s *d* = .12.

H1a and 1b also implied that only girls with a low, as opposed to high, impersonal orientation and hypergendered orientation would differ from boys in the relationship between SEIM use and sexual uncertainty. To test this implication, I conducted slope difference tests for the relationship between SEIM use and sexual uncertainty between boys and girls with low and high levels of impersonal sex orientation and hypergendered orientation. In contrast to Hypothesis 1a, girls with a low impersonal sex orientation did not differ from boys with a high impersonal sex orientation, *t*(1,748) = −0.44, *p* = .66, Cohen’s *d* = .02, nor from boys with a low impersonal sex orientation, *t*(1,748) = −0.53, *p* = .60, Cohen’s *d* = .03. Girls with a high impersonal sex orientation did differ from boys with a high impersonal sex orientation, *t*(1,748) = 1.99, *p* = .046, Cohen’s *d* = .10, but not from boys with a low impersonal sex orientation, *t*(1,748) = 1.76, *p* = .08, Cohen’s *d* = .08.

In line with Hypothesis 1b, girls with a low hypergendered orientation differed from boys with a high hypergendered orientation, *t*(1,748) = 2.10, *p* = .036, Cohen’s *d* = .10, but not from boys with a low hypergendered orientation, *t*(1,748) = 1.70, *p* = .09, Cohen’s *d* = .08. Girls with a high hypergendered orientation did not differ from boys with a high hypergendered orientation, *t*(1,748) = −1.15, *p* = .25, Cohen’s *d* = .06, nor from boys with a low hypergendered orientation, *t*(1,748) = −1.87, *p* = .06, Cohen’s *d* = .09.

### Additional Analyses for Accidental SEIM Use

There was no significant three-way interaction between accidental SEIM use (Wave 1), gender and hypergendered orientation (Wave 1) on sexual uncertainty (Wave 2), *β* = −0.06, *B* = −0.10, *SE* = 0.05, *p* = .05, nor between accidental SEIM use (Wave 1), gender and impersonal sex orientation (Wave 1) on sexual uncertainty (Wave 2), *β* = .05, *B* = .07, *SE* = 0.04, *p* = .12. However, I did find a direct influence of accidental SEIM use on sexual uncertainty, *β* = .07, *B* = .08, *SE* = 0.04, *p* = .03. Moreover, the trend of the three-way interactions for accidental SEIM use seemed to be similar to the three-way interactions with intentional SEIM use.

## Discussion

This study aimed at extending previous research on the sexual uncertainty hypothesis (Peter & Valkenburg, 2010; Sparks, 2013). Specifically, I focused on which types of adolescents are most susceptible to the influence of SEIM on sexual uncertainty. The relationship between SEIM use and sexual uncertainty had previously been expected to differ between girls and boys (Peter & Valkenburg, 2010). I found, however, that the relationship between intentional, but not accidental, SEIM use and sexual uncertainty only held for girls with a low hypergendered orientation and a high impersonal sex orientation. As a result, between-gender differences were conditional on adolescents’ level of hypergendered orientation and impersonal sex orientation: girls with a low hypergendered orientation differed from boys with a high hypergendered orientation and girls with a high impersonal sex orientation differed from boys with a high impersonal sex orientation. Girls with a high hypergendered orientation and a low impersonal sex orientation did not differ from boys. More importantly, the largest differences in the relationship between SEIM use and sexual uncertainty were found between girls with low and high levels of impersonal sex orientation and between girls with low and high levels of hypergendered orientation. It should be noted, however, that the effect sizes of the differences in regression slopes between girls with different levels of hypergendered orientation and impersonal sex orientation were small.

### Implications for Research on SEIM Use and Differential Susceptibility

In contrast to my expectations, I found that girls with *high,* rather than low, levels of impersonal sex orientation were more likely to report more sexual uncertainty in response to higher SEIM use. One explanation of this finding may be that the portrayal of casual sex in SEIM does not depict casual sex in a way that is congruent with girls’ impersonal sex orientation. Specifically, the way that female empowerment and pleasure is depicted in pornography may conflict with girl’s notion of casual sex. Sex in pornography is sometimes depicted in a way that is degrading toward women (e.g., Gorman et al., 2010; McKee, 2005; Monk-Turner & Purcell, 1999). Moreover, sexually explicit material tends to portray male pleasure more often than female pleasure, with men more likely to reach orgasm than women (Bridges et al., 2010; Brosius et al., 1993; Gorman et al., 2010; Klaassen & Peter, 2014). As a result, girls who consider hooking-up and having sex outside a committed relationship pleasurable (i.e., high impersonal sex orientation) may not find this reflected in how casual sex is portrayed in SEIM. Frequent exposure to SEIM may, as a result, increase sexual uncertainty among girls with a high impersonal sex orientation.

Another explanation for this unexpected finding may have to do with the skewness of the measure of impersonal sex orientation. The majority of the sample scored very low on impersonal sex orientation. As a result, high (i.e., one standard deviation above the mean) levels still meant disagreement with the statements referring to an impersonal sex orientation. This means that “high” levels of impersonal sex orientation in the analysis actually refer to rather low impersonal sex orientation. Girls with “high” impersonal sex orientation in the analysis may thus still experience a lack of congruency with the casual sex depicted in sexually explicit internet material. For girls with the lowest levels of impersonal sex orientation girls (“low impersonal sex orientation” in the analysis) depictions of casual sex in sexually explicit internet may be too far removed from their sexual experiences and beliefs for it to have an influence on their certainty about sex.

In line with my expectations, I found that more frequent SEIM use resulted in more sexual uncertainty among girls with low levels of hypergendered orientation. These findings merge with previous research, in which women with low and moderate levels of hypergendered orientation responded with relatively more negative thoughts than positive thoughts to a male-targeted erotic story, and with relatively more positive thoughts than negative thoughts to a female-targeted erotic story (van Oosten et al., 2015). These differences in responses were not found for women with high levels of hypergendered orientation. These previous findings thus also point to a content congruency effect in responses to sexually explicit material that is based on women’s levels of hypergendered orientation. The three-way interaction that was found in the present study complements these previous findings.

The findings of the present study also merge with calls for more attention to differential susceptibility to media effects, both in media effects research in general (Valkenburg & Peter, 2013), and in research on SEIM use in particular (Kingston et al., 2009; Malamuth et al., 2000). With regard to the disposition-content congruency hypothesis (Valkenburg & Peter, 2013), the relationship between SEIM use and sexual uncertainty thus depends on the level of congruency between the sexual content and sexual beliefs, values, and expectations, at least for girls. Future research may investigate whether this relationship further depends on other susceptibility variables stated in the DSMM (i.e., social and developmental susceptibility variables, Valkenburg & Peter, 2013), for both genders. For instance, the relationship between SEIM use and sexual uncertainty may also depend on the congruency between SEIM and adolescents’ social context and physical maturation.

The present study also showed that individual susceptibility variables (i.e., gender and sexual beliefs) can interact in determining the strength of the relation between the use of sexual media and sexual uncertainty. This is especially apparent in the finding that even though sexually explicit material is generally congruent with an impersonal sex orientation, this may not be the case for girls’ impersonal sex orientation. These findings imply that research on content congruency effects should take into account individuals’ sexual beliefs and values, as well as how such beliefs and values are reflected in sexual content for each individual with regard to their gender, but also other characteristics such as age or ethnicity. Although the DSMM does not preclude that dispositional susceptibility variables can interact, it has not explicitly conceptualized such interactions (Valkenburg & Peter, 2013). The present findings thus suggest that the DSMM may be extended by incorporating interactions between dispositional susceptibility variables. Such an extension of the DSMM may be particularly useful in the context of research that has focused on the role of multiple individual difference variables in the emergence of media effects.

Moreover, the finding that within-gender differences in the relationship between sexual media use and sexual uncertainty were most profound for girls suggests that, when it comes to sexual media content, girls may be more susceptible to disposition-content congruency than boys are. One explanation for this greater susceptibility may be that girls often receive contradictory messages about femininity and female sexuality (e.g., “be sexy but not sexual”, Tolman, 2002). As a result, girls may be more preoccupied with figuring out what kind of sexual behavior is expected of them. To this end, they may pay more attention to the specific messages in sexual media content. This may, in turn, increase their susceptibility to influences of sexual media content (Ward, 2003), in particular in relation to their sexual uncertainty. This idea seems to be in line with earlier research on sexual uncertainty in which girls who watched more SEIM were more involved in SEIM, in the sense that they concentrated more on the content and forgot their surroundings while watching the content. This involvement in turn predicted sexual uncertainty (Peter & Valkenburg, 2010).

The findings on within-gender differences seem to be particularly relevant to the confluence model (Kingston et al., 2009; Malamuth et al., 2000, 2012). The present study suggests that the confluence model can be meaningfully extended in at least three ways. First, this study is the first to show that congruency effects that were previously found among adults also occur among adolescents, at least when it comes to the congruency between SEIM and girls’ sexual dispositions. Second, whereas the confluence model has focused on individual susceptibility to effects of sexual material due to high levels of impersonal sex orientation and hypermasculinity, the present study suggests that such susceptibility can in some cases also depend on *low* levels of sexual dispositions. Finally, sexual dispositions can influence the relationship between SEIM use and other outcome variables than sexual aggression (i.e., sexual uncertainty).

Finally, only the influence of intentional SEIM use on sexual uncertainty was moderated by gender and sexual dispositions. The sexually explicit material that adolescents encountered accidentally on the internet increased adolescents’ sexual uncertainty directly, and did not depend on the interplay between gender and sexual dispositions. One explanation for these different findings for intentional and accidental SEIM use may be that adolescents who mostly encounter SEIM accidentally may be unfamiliar with such content and are thus more easily affected by it in their sexual beliefs, regardless of their gender or sexual dispositions. Moreover, when adolescents encounter SEIM accidentally they may process such material less elaborately than when they deliberately look for SEIM. As a result, pre-existing sexual beliefs, such as hypergendered and impersonal sex orientation, are less likely to influence responses to sexual material that adolescents come across accidentally. More research is needed to disentangle the different processes that may occur during intentional and accidental SEIM use.

### Limitations and Conclusion

One limitation of the present study concerns the generalizability of the findings to other cultural contexts. The present study was conducted in the Netherlands, a country that is known for its liberal policy both toward adolescent sexuality and sexually explicit material, and in which boys and girls receive a similar sexual socialization (Schalet, 2000, 2011). Moreover, in cross-cultural research, the Netherlands is considered a feminine society, which is characterized by greater gender equality than masculine societies (Hofstede, 1998, 2001). This greater gender equality may have been related to the absence of between-gender effects for the relationship between SEIM use and sexual uncertainty in previous research (Peter & Valkenburg, 2010), as well as in the present study. Future research should therefore replicate the current findings in more masculine societies, such as the U.S., as well as in countries in which adolescents receive a more gendered sexual socialization.

Another limitation is that the design of the present study does not have the same internal validity as an experimental design. However, manipulating SEIM use among adolescents in a study is ethically very problematic. Future research could, however, experimentally test the relationships found in this study among young adults. Some studies have already found comparable results with experimental designs among adults. For instance, as mentioned before, women with low and moderate levels of hypergendered orientation have been shown to respond more critically toward sexually explicit material than women with high levels of hypergendered orientation (van Oosten et al., 2015), and women become differently involved with a sexual character, based on their levels of impersonal sex orientation (Boot et al., 2014).

When replicating the present study in an experimental design among adults, however, it may be difficult to generalize these findings to adolescents. Adolescence is a period characterized by the development of the sexual self (e.g., Steinberg, 2008) and high sexual curiosity (Savin-Williams & Diamond, 2004). It thus seems likely that adolescents are more susceptible to effects on their sexual selves, and thus sexual uncertainty, than adults. That said, the longitudinal design, in which I also controlled for the autoregressive relationship between sexual uncertainty at Wave 1 and Wave 2, enables drawing some conclusions about the causal relationships between disposition-content congruency and sexual uncertainty over time.

In conclusion, the present study shows that the relationship between SEIM use and sexual uncertainty depends on sexual dispositions that differ within gender. Whereas it was previously thought that this relationship would be stronger for girls than for boys, this research showed that such susceptibility applies only to a subgroup of girls. This also implies that research on sexual media effects should take both between-gender differences and within-gender differences in sexual dispositions into account. Only then can we increase our understanding of who is susceptible to the effects of SEIM use on sexual outcomes, such as sexual uncertainty.
